# Severity of Repetitive Mild Traumatic Brain Injury Depends on Microglial Heme Oxygenase‐1 and Carbon Monoxide

**DOI:** 10.1111/ejn.16666

**Published:** 2025-01-22

**Authors:** Sandra Kaiser, Anna Fritsch, Lena Jakob, Nils Schallner

**Affiliations:** ^1^ Department of Anesthesiology & Critical Care Medical Center—University of Freiburg Freiburg Germany; ^2^ Faculty of Medicine University of Freiburg Freiburg Germany

**Keywords:** astrocytes, carbon monoxide, heme oxygenase‐1, microglia, neuroinflammation, traumatic brain injury

## Abstract

Traumatic brain injury is one of the most common cerebral incidences worldwide. Repetitive mild traumatic brain injuries occurring, for example, in athletes or victims of abuse, can cause chronic neurodegeneration due to neuroinflammation, in which the crosstalk between reactive astrocytes and activated microglia is crucial for modulating neuronal damage. The inducible enzyme heme oxygenase‐1 and its product carbon monoxide are known to be ascribed neuroprotective and anti‐inflammatory properties. We caused repetitive mild traumatic brain injuries in wild‐type mice compared to mice without microglial heme oxygenase‐1 expression. Additionally, mice were treated daily with either air or carbon monoxide exogenously. In wild‐type mice, we observed enhanced microglia activation and astrogliosis as well as vasodilation after repetitive trauma. In heme oxygenase‐1 knockout mice, we observed enhanced activation of microglia and astrocytes at baseline pretrauma with a lack of an adequate inflammatory response to repetitive injury. However, the knockout led to enhanced NF‐κB and IFNγ expression in the post‐trauma period. Carbon monoxide exerted neuroprotection, as suggested by reduced wake‐up times in mice and by beneficially altering inflammation post‐traumatic brain injury. This study further underlines the crucial role of the heme oxygenase‐1/carbon monoxide system in the modulation of neuronal damage and the associated neuroinflammatory response after repetitive traumatic brain injury.

## Graphical abstract

Activated microglia enhanced NF‐κB mRNA expression, cytokine IFNγ release, vasodilation and astrogliosis, which were modulated by microglial heme oxygenase‐1 expression. Exogenous carbon monoxide treatment led to a reduction in the inflammation response (abbreviations: rmTBI: repetitive mild traumatic brain injuries; HO‐1: heme oxygenase‐1; CO: carbon monoxide). In the work “Severity of Repetitive Mild Traumatic Brain Injury Depends on Microglial Heme Oxygenase‐1 and Carbon Monoxide” (Sandra Kaiser, Anna Fritsch, Lena Jakob and Nils Schallner*), repetitive mild traumatic brain injury (rmTBI) activated microglia in a mouse model in a heme oxygenase‐1 depended on manner. Activated microglia enhanced astrogliosis, inflammation, vasodilation and cytokine release which were modulated by microglial heme oxygenase‐1 expression. Exogenous carbon monoxide treatment led to a reduction in the inflammation response and enhanced the recovery in this rmTBI model.

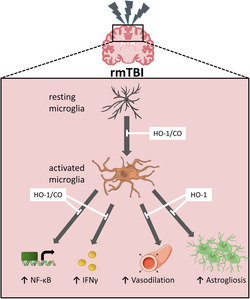

**Abbreviations**
°C: Celsius
μg: μgram
μL: μliter
μm: μmeter
CBA: cytometric bead array
cDNA: complementary deoxyribonucleic acid
cm: centimetre
CO: carbon monoxide
DAPI: 4′,6‐diamidino‐2‐phenylindole
DFn: degrees of freedom for the numerator
DFd: degrees of freedom for the denominator
fl: flox
g: gram
GFAP: glial fibrillary acidic protein
Hmox1: heme oxygenase‐1 (mouse strain genetic information)
HO‐1: heme oxygenase‐1 (gene/protein)
Iba‐1: ionized calcium‐binding adapter molecule 1
kg: kilogram
KO: knockout
IFNγ: interferon gamma
LR: lumen radius
Lyz: lysozyme
MCA: middle cerebral artery
mg: milligram
min: minute
mL: millilitre
ns: nonsignificant
mTBI: mild traumatic brain injury
NF‐κB: nuclear factor kappa B
PBS: phosphate buffered saline
PCR: polymerase chain reaction
PFA: paraformaldehyde
pg: picogram
ppm: parts per million
rmTBI: repetitive traumatic brain injury
RNA: ribonucleic acid
Rplp0: ribosomal protein, large, P0
SAH: subarachnoidal haemorrhage
SD: standard deviation
TBI: traumatic brain injury
TBS: tris‐buffered saline
W: watt
WT: wall thickness

## 1 Introduction

Traumatic brain injury (TBI) is one of the most common cranial pathologies globally. Around 69 million people suffer from TBI each year. The majority (> 80%) of these are mild traumatic brain injury (mTBI) (Dewan et al. 2018). The exact number is predicted to be higher as minor injuries may not be recorded when people are not hospitalized. Repetitive mild traumatic brain injuries (rmTBIs) can not only cause acute cognitive impairments but also trigger chronic neurodegenerative disorders including Alzheimer's (Dams‐O'Connor et al. 2016), Parkinson's disease (Gardner et al. 2018) and dementia (Lee et al. 2013). rmTBIs are known to occur among athletes in contact sports, military personnel, or victims of domestic violence. The cumulative impact of repetitive trauma raises important health concerns. Chronic inflammation due to rmTBI causes gradually progressing neurodegeneration, especially in the cerebral cortex and hippocampus (Postolache et al. 2020).

Vasoregulation strongly influences the outcome of patients after TBI, as it is crucial for effective brain perfusion. In mTBI, proper vasoregulation enhances recovery and reduces symptoms like headaches. Post‐TBI inflammation can lead to the widening of blood vessels. This increases blood flow, resulting in edema and higher intracranial pressure, which in turn reduces oxygen delivery to brain cells, ultimately causing neuronal damage (Schwarzmaier et al. 2015; Laird et al. 2014). In cases of rmTBIs, the effects tend to accumulate, leading to an expected worsening of outcomes. However, the effects of rmTBI in humans remain unexplored to a certain extent, as these patients are less likely to be hospitalized compared to those with more severe TBIs. Nonetheless, several animal studies using rmTBI models have demonstrated increased inflammation and notable morphological changes (Hoogenboom, Branch, and Lipton 2019).

Upon injury, microglia, as part of the immune system of the central nervous system, shift into an activated state, characterized by morphological hypertrophy. They play a key role in modulating neuronal damage through the release of cytokines such as the proinflammatory cytokine IFNγ (Shields, Haque, and Banik 2020; Donat et al. 2017; Fenn et al. 2014). It has been reported that the NF‐κB signalling pathway plays a central role in microglia activation (Zhuang et al. 2017; Li et al. 2019). NF‐κB, as a key transcription factor, is a crucial regulator of inflammation response in TBI (Liu et al. 2024). Interestingly, the stress‐induced molecule heme oxygenase‐1 (HO‐1) has been reported to inhibit the nuclear translocation of NF‐κB (Bellezza et al. 2012). This inhibition reduces the proinflammatory effects of the transcription factor, including cytokine release. Induction of HO‐1 promotes protection in different organ systems, including the central nervous system (Zhang et al. 2012; Saleem et al. 2008; Wang, Xu, and Zhu 2012). The inducible HO‐1 enzyme degrades heme into iron, biliverdin and carbon monoxide (CO). Several animal studies on TBI have demonstrated the protective role of HO‐1, primarily attributed to its antioxidant and anti‐inflammatory effects (Zhu et al. 2018; Dai et al. 2018; Yang et al. 2016; Shu et al. 2016). More precisely, the transcription factor NRF2 leads to enhanced HO‐1 expression, favouring better outcomes even though the HO‐1 mechanisms still need to be further studied. Our previously published data reveal a novel role for microglial HO‐1 in subarachnoid haemorrhage (SAH) (Kaiser et al. 2019; Schallner et al. 2015; Kaiser et al. 2024; Henrich et al. 2023). We demonstrated that HO‐1 expression in microglia modulated the blood clearance and the production of endogenous CO. Additionally, we displayed the neuroprotective and anti‐inflammatory effects of CO.

Alongside microglia, astrocytes, a subtype of glial cells, play the main role in injury response post‐TBI (Karve, Taylor, and Crack 2016). In healthy tissue, astrocytes contribute essentially to maintaining the optimal conditions for neuronal function and survival (Valles et al. 2023). Post‐TBI, the proinflammatory state of microglia promotes astrocyte activation and astrogliosis with an increase of reactive astrocytes at the inflammation site (Zheng et al. 2022; Sofroniew and Vinters 2010; Witcher et al. 2018). Previous reports highlight the critical role of crosstalk between reactive astrocytes and microglia in modulating inflammation, neuronal damage and recovery (Liddelow et al. 2017; Henrik Heiland et al. 2019).

In the study presented herein, we utilized an rmTBI mouse model to further explore the role of HO‐1 in modulating neuroinflammation and recovery postinjury. We postulate that the induced inflammatory response depends on microglial HO‐1 expression and that the protective effects of HO‐1 are, at least in part, mediated by CO. We also hypothesize that exogenous CO holds a therapeutic potential in the treatment of TBI. Low therapeutic doses of CO have been investigated in several human studies and diseases (Bansal et al. 2024), although its clinical application remains under development. Our study is aimed at providing deeper insight into the role of the HO‐1/CO system in inflammations post‐TBI. This knowledge could serve as a foundation for further studies exploring the clinical significance of the HO‐1/CO pathway and its modulation to reduce the long‐term detrimental effects of repetitive brain injury.

## 2 Materials and Methods

### 2.1 Animals and Anaesthesia

Cell‐type‐specific HO‐1 knockout (KO) in myeloid cells, including microglia (*LyzMCre‐Hmox1* ^*fl/fl*^) was achieved by crossing *Hmox1* ^*fl/fl*^ mice (RIKEN Bio Resource Center, RBRC03163; RRID:IMSR_RBRC03163) with mice expressing Cre recombinase under the lysozyme (Lyz) promoter (Jackson Laboratory, #004781; RRID:IMSR_JAX:004781). *Hmox1* ^*fl/fl*^ microglia were used as wild‐type controls for all in vitro studies. For all experiments, male mice (adult 8–14 weeks) were used. Animals were fed with a standard rodent diet ad libitum while kept on a 12‐h light/12‐h dark cycle. Thirty minutes prior to intervention, buprenorphine (50 μg/kg) was applied subcutaneously to treat potential pain. Euthanasia of the narcotized mice took place by trans‐cardiac perfusion after anaesthesia with ketamine (100 mg/kg) and xylazine (5 mg/kg).

### 2.2 TBI Model

An impact device, as previously described in the literature (Meehan et al. 2012), was used to induce rmTBI. The device consists of an 88 cm long guiding tube and a metal cylinder with a diameter of 1.27 cm, weighing 54 g. This setup delivers a very mild impact that does not cause fractures to the skull and no brain bleeding, enabling the induction of a focal blunt injury.

**SCHEME 1:**
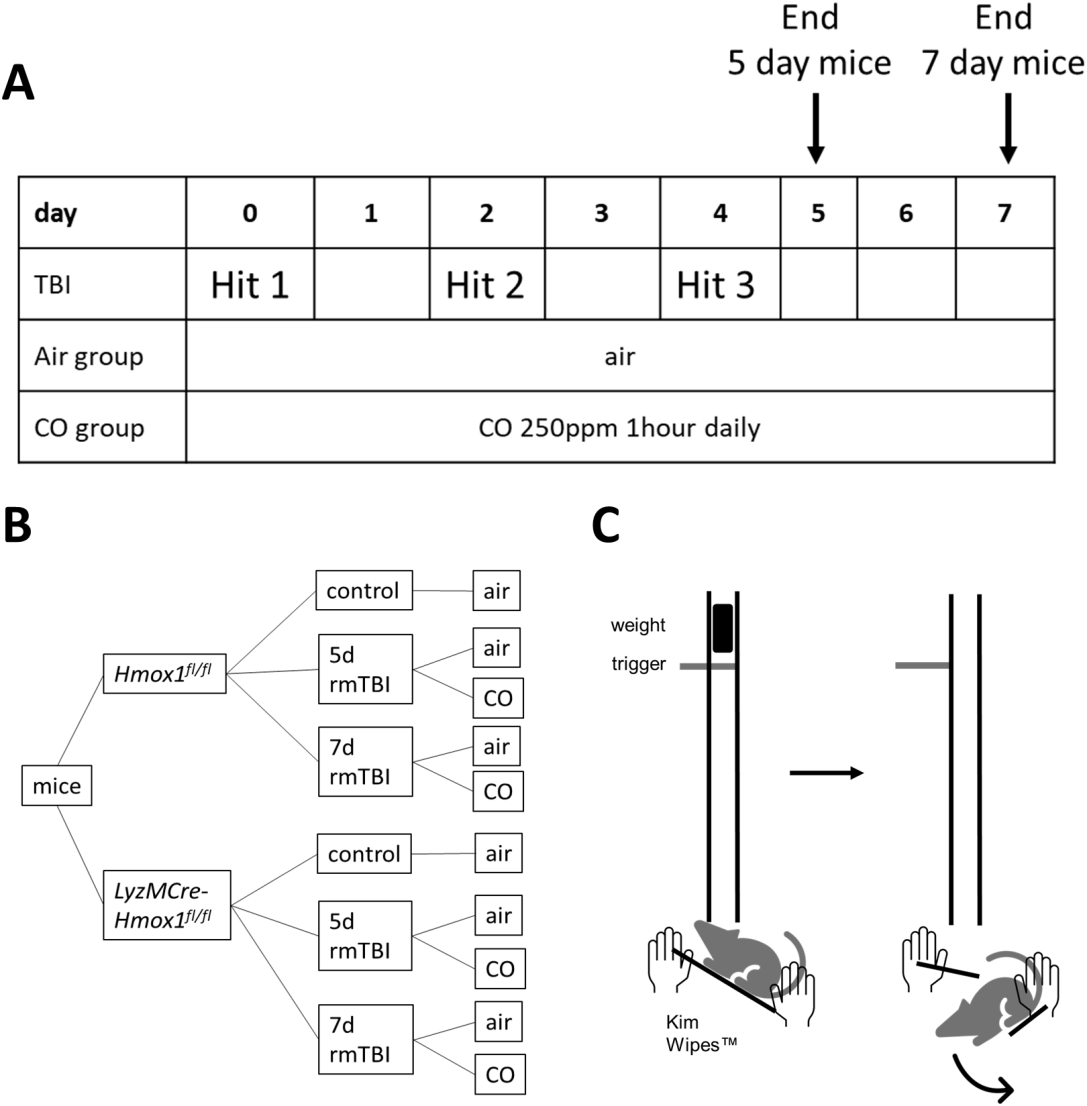
Schematic representation of the mouse experiments. (A) Graphical timeline. (B) Overview of mouse groups. (C) Schematic representation of the TBI model. In the experimental setup, an anaesthetised mouse is positioned on a layer of Kimwipes for manual stabilization before being placed beneath the metal cylinder. By releasing the trigger, the calibrated weight falls freely, inducing a mild traumatic brain injury (TBI) in the mouse. The impact tears the Kimwipes layer, and as the mouse remains secured by holding onto the tail, it swings backward, simulating a rebound motion. The illustration was created using Microsoft PowerPoint. TBI: traumatic brain injury; rmTBI: repetitive traumatic brain injury; CO: carbon monoxide; ppm: parts per million.

After inhalational anaesthesia with isoflurane (4% in pure oxygen), the mouse was positioned at a 45° angle with its head centrally beneath the guiding tube on Kimwipes. The impact caused the cloth to tear, allowing the mouse to swing freely, simulating a rebound motion (Scheme 1). The first steps on all four limbs after the impact were defined as the waking‐up‐recovery time.

The rmTBI was performed during the second hour of the light phase on three separate days (Day 0, Day 2 and Day 4), with a 48‐h interval between each impact. Organ extraction, followed by subsequent analyses, took place 24 h (5 days after the first mTBI) or 72 h (7 days after the first mTBI) after the last trauma (Scheme 1). Mice in the control group did not undergo rmTBI. They were anaesthetised on Day 0 without impact, and organ extraction was performed on Day 1.

Thirty minutes before the trauma, analgesia was administered with buprenorphine (50 μg/kg), continued every 6 h until 24 h after the final trauma. For nightly buprenorphine doses, the way of administration was switched to water supplementation (0.3 mg per 30 mL water + 2 mL 5% glucose). In the 7‐day group, pain medication therapy was continued with carprofen (10 mg/kg) every 12 h for an additional 48 h.

### 2.3 CO Gas Treatment

After mTBI, animals were randomly assigned to either receive treatment with CO or synthetic air for 1 h. CO exposure was done in a custom‐made chamber. Animals had free access to food and water during the treatment. Premixed air with 250 ppm CO was used. This specific CO dose is well‐tolerated and has no adverse effects on the well‐being of the mice. It is a well‐established dose in animal studies and has been previously reported in the literature (Kaiser et al. 2024; Henrich et al. 2023; Kaiser et al. 2020). CO concentration was monitored continuously throughout the exposure with an infrared gas analyser. Treatment was started immediately after the first mTBI and repeated every 24 h until the end of the experimental period.

### 2.4 Cytometric Bead Array (CBA Assay)

Peripheral blood was collected by heart puncture and transferred into heparin‐coated tubes. After centrifugation, plasma was analysed using bead‐based flow cytometry as the manufacturer instructed (BD 552364, Mouse Inflammation Kit) on a BD Fortessa flow cytometer. Data were processed with the FCAP Array Software.

### 2.5 Haematoxylin/Eosin Staining and Evaluation of Cerebral Vasoregulation

At the end of the mouse experiment, animals were deeply anaesthetised with ketamine and xylazine and transcardially perfused with TBS followed by paraformaldehyde (PFA) 4%. Brains were removed and fixed in PFA 4% for 18 h. After cryoprotection in sucrose, brains were frozen, cut into 8 μm sections and stained with haematoxylin/eosin. Representative digital images of three consecutive middle cerebral arteries (MCAs) cross‐sections from each animal were obtained, and the lumen radius/wall thickness ratio was quantified to assess vasoregulation using ImageJ.

### 2.6 Immunohistochemistry

Frozen brains were cut into 8 μm serial coronal sections. The glass slides with brain sections were heated in 1× citrate buffer pH 6 (Zytomed System GmbH, K035) in a microwave (3 × 5 min 800 W). After a 20‐min cooldown, permeabilization was done with 0.1% Tween/PBS (Iba‐1) or 0.1% Triton X100/PBS (glial fibrillary acidic protein [GFAP]) for 10 min at room temperature. Slides were then blocked in 10% donkey serum/PBS for 30 min at room temperature. Staining was performed using primary antibodies against Iba‐1 (Abcam Cat# ab5076, RRID:AB_2224402; 1:200) or GFAP (Abcam Cat# ab7260, RRID:AB_305808; 1:500) at 4°C overnight. Then, for fluorescent imaging, sections were incubated with the corresponding secondary antibody 1:1000 for 1 h (for Iba‐1 anti‐goat Alexa Fluor 488, Abcam Cat# ab150129, RRID:AB_2687506, for GFAP anti‐rabbit Alexa Fluor 488, Abcam Cat# ab150073, RRID:AB_2636877). Nuclear counterstaining was done with DAPI (4′,6‐diamidino‐2‐phenylindole) (5 min), and cover slides were added with mounting medium (Agilent Dako, S302380–2). Slides were examined under a bright field and fluorescence microscope (Zeiss AxioObserver Invert, Plan‐Apochromat 20x/0.8 fluorescence and EC Plan‐Neofluar 40x/0.75 Ph2 for bright field). To achieve appropriate consistency in the quantification of cells, from each area of interest, four images were obtained of three mice per group, resulting in 12 analysed images per group. Iba‐1 was used as a microglia marker and GFAP as an astrocyte marker. For analysis of gliosis, GFAP‐positive cells were counted in relation to counts of DAPI signals representing whole cell numbers. Microscopic data of GFAP‐positive cell numbers were normalized to DAPI, as the selected sections, despite their identical size, differ in general cell counts. Activated microglia are characterized by larger cell bodies with retracted extensions while resting microglia show smaller cell bodies with extended ramifications. Therefore, we measured the soma size of Iba‐1 positive microglia as a hallmark of activation by area with ZEN (blue edition) software from Carl Zeiss Microscopy (ZEISS ZEN Microscopy Software (RRID:SCR_013672)).

### 2.7 Gene Expression Analyses From Tissue Samples

Upon removal from the mice, the brain was transferred to a sagittal 1 mm matrix. The cortex was removed from slices and snap‐frozen in liquid nitrogen. Upon thawing, samples were immediately homogenized in TRIzol and further processed for RNA purification. RNA purification was done using spin columns (RNEasy mini kit, Qiagen). cDNA libraries were acquired by reverse transcription (iScript cDNA synthesis kit, BioRad). Gene expression was analysed by real‐time PCR (SYBR green master mix, Agilent Technologies) with Rplp0 as a reference gene. Primers were ordered from Eurofins Genomics.

The primer sequences are as follows:

1 NF‐κB

Forward: GCTGCCAAAGAAGGACACGACA.

Reverse: GGCAGGCTATTGCTCATCACAG.

2 Rplp0

Forward: GAGGAATCAGATGAGGATATGGGA.

Reverse: AAGCAGGCTGACTTGGTTGC.

### 2.8 Statistics

Data were analysed with a computerized statistical program (GraphPad Prism (RRID:SCR_002798) Version 10). Results are presented either as columns or as box blots (whiskers indicate minimum and maximum; the line in the box marks the median; the “+” in the box shows the position of the mean). Two groups were compared with Student's *t*‐test. For relative comparison versus an averaged control group, one‐column statistics with one‐sample *t*‐tests versus 1 were used (mRNA expression). Several groups were compared either by mixed‐effects analysis (wake‐up recovery time) or one‐way ANOVA. The statistical tests were performed in a two‐tailed approach. A *p*‐value smaller than 0.05 was considered to be statistically significant.

### 2.9 Study Approval

All procedures involving the animals were approved by the Committee of Animal Care of the University of Freiburg (Permit No. G20–159) and conducted in accordance with the ARRIVE guidelines and the Directive 2010/63/EU by the European Union.

## 3 Results

### 3.1 rmTBI Mouse Model Showed Enhanced Inflammation in the Brain

**FIGURE 1:**
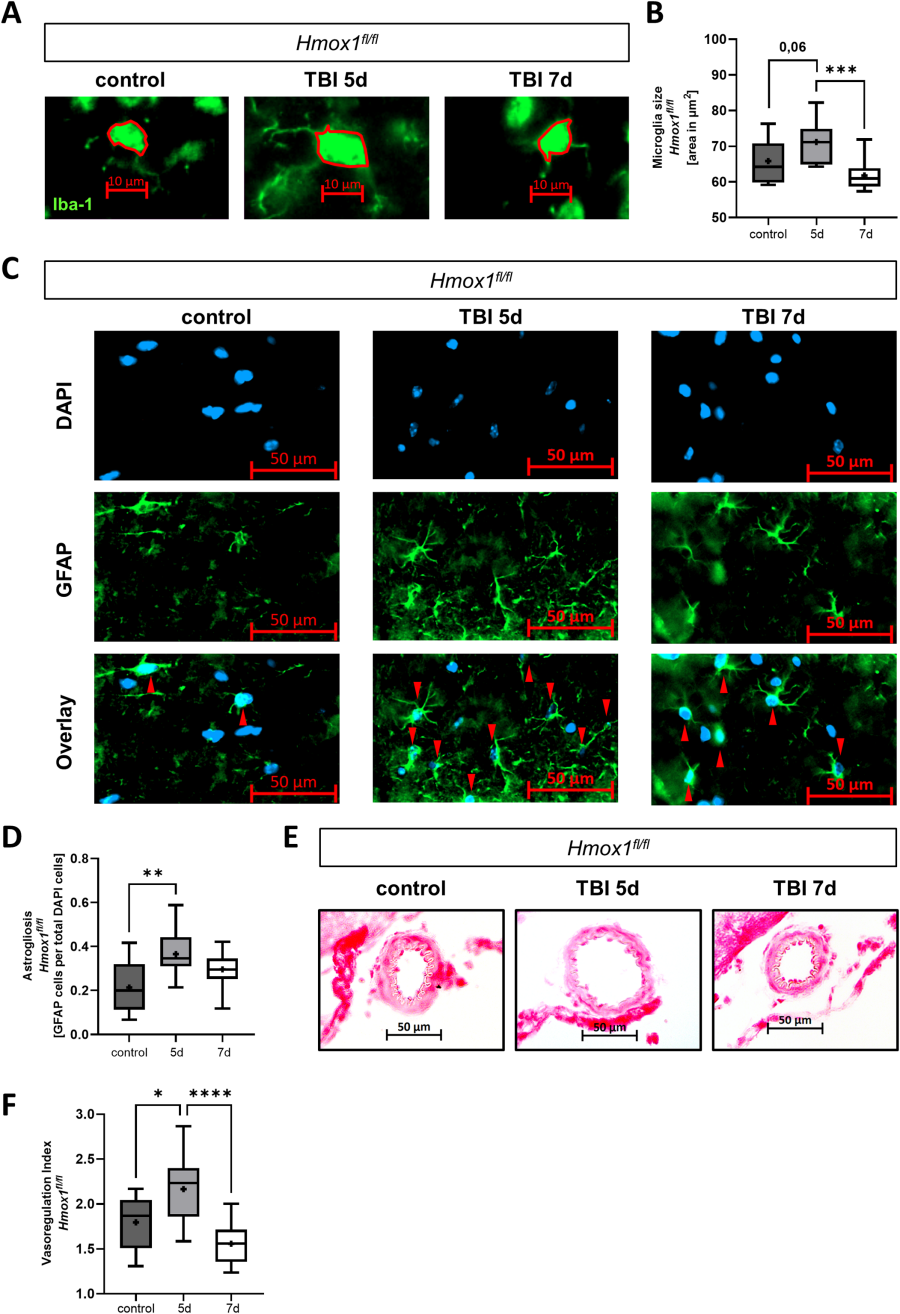
Enhanced inflammation in the mouse brain post‐repetitive mild traumatic brain injury (rmTBI). (A) Representative microglia in the cortex of *Hmox1* ^*fl/fl*^ mice; measurement of soma area; scale bars 10 μm, control without TBI and 5 or 7 days post first impact of rmTBI. (B) Cortex microglia soma size (representative images in (A)), *n* = 12 analysed images per group; *Hmox1* ^*fl/fl*^ control *p* = 0.0006 5 days versus 7 days and *p* = 0.0624 control versus 5 days (*F* (DFn, DFd) (2, 33) = 8.675). (C) Representative images of astrocyte counting in the cortex of *Hmox1* ^*fl/fl*^ mice; green GFAP protein staining and blue DAPI DNA stain; red arrows mark double positive areas; scale bars 50 μm; control without TBI and 5 or 7 days post first impact of rmTBI. (D) Quantification of the number of astrocytes in the cortex of *Hmox1* ^*fl/fl*^ mice; represented as GFAP‐DAPI‐double‐positive cells per total DAPI count; *n* = 12 analysed images; *p* = 0.0017 control versus 5 days (*F* (DFn, DFd) (2, 33) = 7.238). (E, F) Analysis of vasoregulation on haematoxylin/eosin–stained cross‐sections of the middle cerebral artery (MCA) of *Hmox1* ^*fl/fl*^ mice; control without TBI and 5 or 7 days post first impact of rmTBI. (E) Representative haematoxylin/eosin–stained cross‐sections of the MCA. Scale bars represent 50 μm. (F) Quantification of the vasoregulation index (lumen radius [LR]/wall thickness [WT]) in the MCA. *n* = 11–15 analysed images per group; *p* = 0.0164 control versus 5 days; *p* = <0.0001 5 days versus 7 days (*F* (DFn, DFd) (2, 35) = 12.51). Statistical analysis: ordinary one‐way ANOVA with multiple comparison tests (Tukey's). Results were presented as box blot (whiskers indicate minimum and maximum, the line in the box marks the median and the “+” in the box shows the position of the mean); statistically significant values were defined as *p* ≤ 0.05 (*p ≤ 0.05; **p ≤ 0.01, ***p ≤ 0.001 and **** *p* ≤ 0.0001); abbreviations: rmTBI = repetitive mild traumatic brain injury, d = day, μm = μmeter.

To further understand the mechanisms of the damage response in the central nervous system, we investigated histological brain sections of *Hmox1* ^*fl/fl*^ mice post‐rmTBI. Therefore, we compared control mice without rmTBI to mice at Days 5 and 7 post‐rmTBI. Five days after the first impact of rmTBI, cortical microglia exhibited the highest activation state compared to Day 7, as indicated by an increase in soma size (Figure 1A,B). This higher state of inflammation was also confirmed by an increased number of astrocytes in the cortex on Day 5, as a sign of astrogliosis (Figure 1C,D). Moreover, TBI is known to disrupt cerebral autoregulation, leading to vasodilation of brain vessels (Schwarzmaier et al. 2015; Kinoshita 2016). Thus, we examined the state of vasoregulation in this rmTBI model. We could observe an increased vasodilation on Day 5 post‐rmTBI compared to control mice without rmTBI. Seven days post‐rmTBI, there was no significant increase in vasodilation compared to control mice (Figure 1E,F). Taken together, we see an enhanced state of inflammation in *Hmox1* ^*fl/fl*^ mice on Day 5 post‐rmTBI, which decreased toward baseline 7 days post‐rmTBI.

### 3.2 A rmTBI Mouse Model With Microglial HO‐1 KO Showed Altered Impact Response in the Brain Post‐rmTBI

**FIGURE 2:**
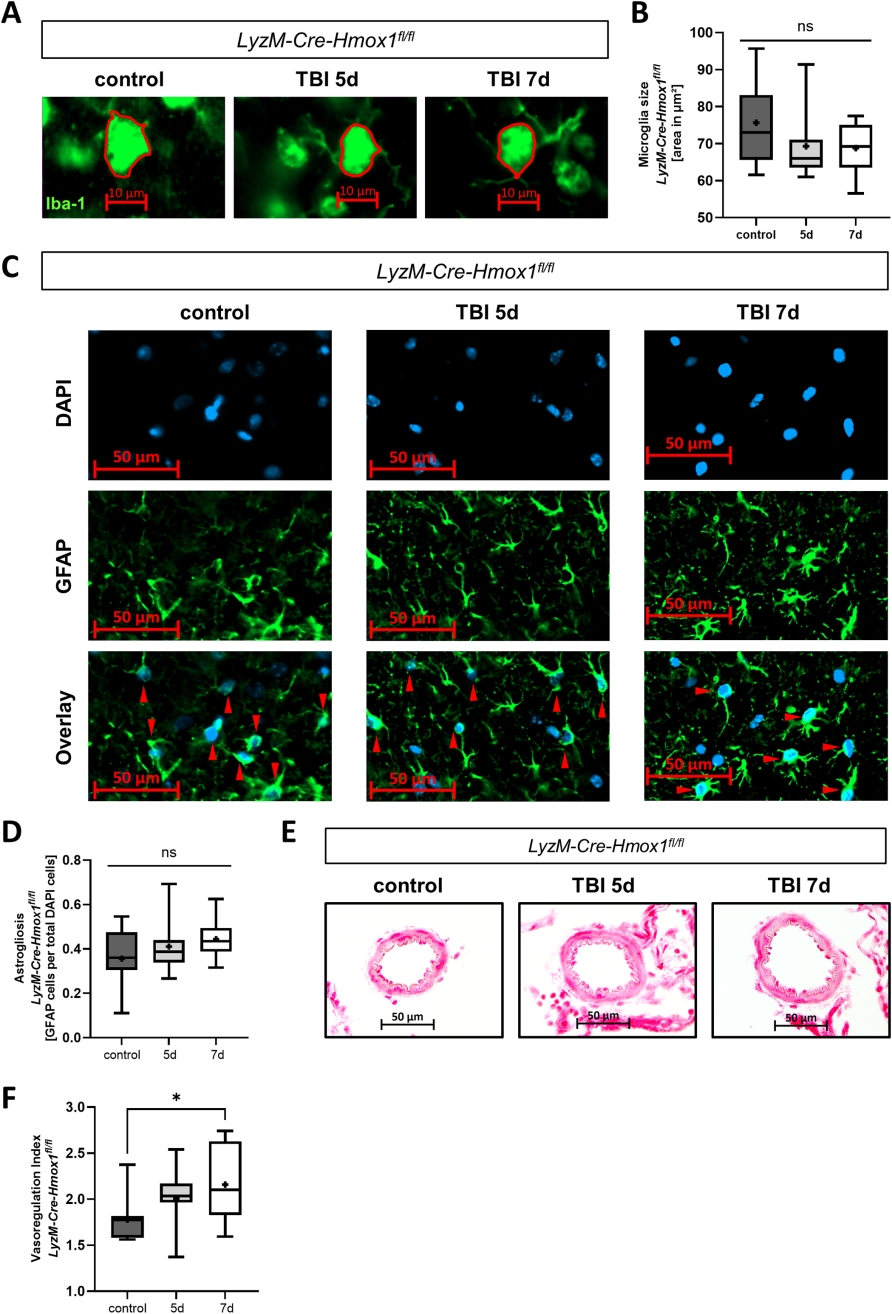
Altered inflammation in the brain from a mouse model with microglial heme oxygenase‐1 (HO‐1) knockout (KO) post‐repetitive mild traumatic brain injury (rmTBI). (A) Representative microglia in the cortex of *LyzM‐Cre‐Hmox1* ^*fl/fl*^ mice; green Iba‐1 protein staining; measurement of soma area; scale bars 10 μm, control without TBI and 5 or 7 days post first impact of rmTBI. (B) Cortex microglia soma size (representative images in (A)), *n* = 12 analysed images per group; *p* = ns (*F* (DFn, DFd) (2, 32) = 2.013). (C) Representative images of astrocyte counting in the cortex of *LyzM‐Cre‐Hmox1* ^*fl/fl*^ mice; green GFAP protein staining and blue DAPI DNA stain; red arrows mark double positive areas; scale bars 50 μm; control without TBI and 5 or 7 days post first impact of rmTBI. (D) Quantification of the number of astrocytes in the cortex of *LyzM‐Cre‐Hmox1* ^*fl/fl*^ mice; represented as GFAP‐DAPI‐double‐positive cells per total DAPI count; *n* = 12 analysed images; *p* = ns (*F* (DFn, DFd) (2, 33) = 1.926). (E, F) Analysis of vasoregulation on haematoxylin/eosin–stained cross‐sections of the middle cerebral artery (MCA) of *LyzM‐Cre‐Hmox1* ^*fl/fl*^ mice control without TBI and 5 or 7 days post first impact of rmTBI. (E) Representative haematoxylin/eosin–stained cross‐sections of the MCA. Scale bars represent 50 μm. *n* = 12 analysed images. (F) Quantification of the vasoregulation index (lumen radius [LR]/wall thickness [WT]) in the MCA. *n* = 12–16 analysed images per group; *p* = 0.0336 control versus 7 days (*F* (DFn, DFd) (2, 34) = 3.451). Statistical analysis: ordinary one‐way ANOVA with multiple comparison tests (Tukey's). Results were presented as box blot (whiskers indicate minimum and maximum, the line in the box marks the median and the “+” in the box shows the position of the mean); statistically significant values were defined as *p* ≤ 0.05 (*p ≤ 0.05); abbreviations: rmTBI = repetitive mild traumatic brain injury, d = day, μm = μmeter.

In a second step, we investigated the influence of microglial HO‐1 post‐rmTBI on damage response in *LyzM‐Cre‐Hmox1* ^*fl/fl*^ mice with HO‐1 KO in microglia. Interestingly, rmTBI did not affect microglia size in the HO‐1 KO mice on Days 5 and 7 post‐rmTBI (Figure 2A,B). Also, astrocyte numbers in the cortex remained constant 5 and 7 days post‐rmTBI (Figure 2C,D). However, an increase in vasodilation was observed on Day 7 but not on Day 5 post‐rmTBI (Figure 2E,F). Thus, microglial HO‐1 KO leads to an altered, blunted activation and damage response following rmTBI.

### 3.3 Altered Inflammation Response in Mice Without Microglial HO‐1 Is Caused by Elevated Basal State in KO Mice

**FIGURE 3:**
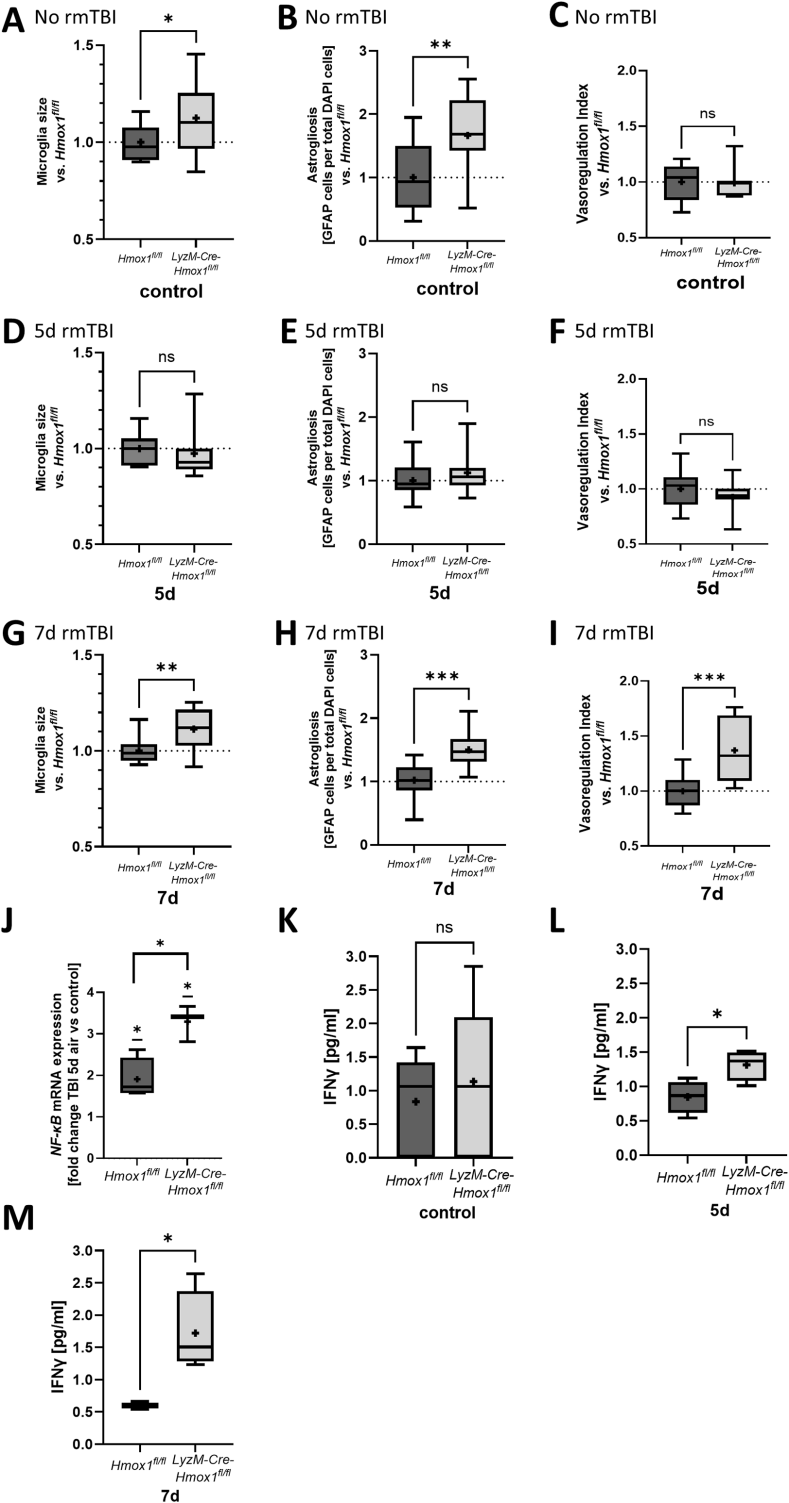
Altered inflammation response in mice without microglial heme oxygenase‐1 (HO‐1) caused by elevated basal state in the knockout (KO) mice. (A–I) Comparison of *LyzM‐Cre‐Hmox1* ^*fl/fl*^ data with *Hmox1* ^*fl/fl*^ data from the repetitive mild traumatic brain injury (rmTBI) model; control without TBI and 5 or 7 days post first impact of rmTBI; calculated by the division *LyzM‐Cre‐Hmox1* ^*fl/fl*^/*Hmox1* ^*fl/fl*^ mice. (A, D, G) Cortex microglia soma size; *n* = 12 analysed images per group. (A) *p* = 0.048 (*F* (DFn, DFd) (11, 11) = 3.913), (D) *p* = ns (*F* (DFn, DFd) (11, 11) = 2.092) and (G) *p* = 0.0049 (*F* (DFn, DFd) (11, 11) = 2.885). (B, E, H) Quantification of the number of astrocytes in the cortex; measured as GFAP‐DAPI‐double‐positive cells per total DAPI count; *n* = 12 analysed images; (B) *p* = 0.0096 (*F* (DFn, DFd) (11, 11) = 1.462), (E) *p* = ns (*F* (DFn, DFd) (11, 11) = 1.194) and (H) *p* = 0.0002 (*F* (DFn, DFd) (11, 12) = 1.075). (C, F, I) Quantification of the vasoregulation index (lumen radius [LR]/wall thickness [WT]) in the MCA. *n* = 11–16 analysed images per group; (C) *p* = ns (*F* (DFn, DFd) (10, 8) = 1.461), (F) *p* = ns (*F* (DFn, DFd) (14, 15) = 1.713) and (I) *p* = 0.0006 (*F* (DFn, DFd) (11, 11) = 3.727). (J) NF‐κB mRNA level in the cortex of *Hmox1* ^*fl/fl*^ and *LyzM‐Cre‐Hmox1* ^*fl/fl*^ mice 5 days post first impact of rmTBI demonstrated as fold change versus mice without TBI; *n* = 3–4 mice; *p* = 0.0118 *Hmox1* ^*fl/fl*^ and *LyzM‐Cre‐Hmox1* ^*fl/fl*^; *p* = 0.0343 (*F* (DFn, DFd) (3, 2) = 1.260). *Hmox1* ^*fl/fl*^ versus 1 (df = 3); *p* = 0.0119 *LyzM‐Cre‐Hmox1* ^*fl/fl*^ versus 1 (df = 2). (K–M) Flow cytometric analysis of cytokine IFNγ protein level in peripheral blood of *Hmox1* ^*fl/fl*^ and *LyzM‐Cre‐Hmox1* ^*fl/fl*^ rmTBI mice; control without TBI (K) and 5 (L) or 7 (M) days post first impact of rmTBI; *n* = 6–10 (K); *n* = 4 (L, M); (K) *p* = ns (*F* (DFn, DFd) (5, 9) = 2.647); (L) *p* = 0.0288 (*F* (DFn, DFd) (3, 3) = 1.139); (M) *p* = 0.03 (*F* (DFn, DFd) (3, 2) = 109.7). Results presented as box blot (whiskers indicate minimum and maximum, the line in the box marks the median and the “+” in the box shows the position of the mean) (A–I) and as bar (mean with SD) (J–M); statistical analysis used unpaired *t*‐test (A–M) and one sample *t*‐test (J). Statistically significant values were defined as *p* ≤ 0.05 (*p ≤ 0.05; **p ≤ 0.01; ***p ≤ 0.001); abbreviations: rmTBI = repetitive mild traumatic brain injury, d = day; pg/mL = picogram per millilitre.

*LyzM‐Cre‐Hmox1* ^*fl/fl*^ mice with microglial HO‐1 KO, which did not undergo rmTBI, showed an increase in microglial size and astrogliosis but not in vasodilation compared to *Hmox1* ^*fl/fl*^ control mice (Figure 3A–C). This direct comparison of mice deficient for microglial HO‐1 with *Hmox1* ^*fl/fl*^ mice points toward an elevated basal state of activation in HO‐1 KO mice. Microglia size, astrocyte numbers, and vasodilation did not differ 5 days post‐rmTBI, demonstrating a comparable state of inflammation (Figure 3D–F). Seven days post‐rmTBI, KO mice had larger microglia, a higher number of astrocytes in the cortex and elevated vasodilation compared to *Hmox1* ^*fl/fl*^, indicating sustained activation of the microglia‐astrocyte axis in mice lacking microglial HO‐1 (Figure 3G–I). To verify the elevated basal state in HO‐1 KO mice and the enhanced inflammation post‐rmTBI in both genotypes, the expression of the transcription factor NF‐κB, a critical mediator of proinflammatory gene expression and itself upregulated in inflammation, was measured in the brain tissue. While in both *Hmox1* ^*fl/fl*^ and *LyzM‐Cre‐Hmox1* ^*fl/fl*^ mice, NF‐κB mRNA expression was elevated in the cortex 5 days post‐rmTBI, the KO mice showed an even higher inducibility (Figure 3J). Moreover, the brain injury marker S100B, primarily expressed in astrocytes, and the global HO‐1 expression in the cortex were analysed. Both S100B and HO‐1 mRNA expression levels were elevated post‐TBI to a similar extent in *Hmox1* ^*fl/fl*^ and *LyzM‐Cre‐Hmox1* ^*fl/fl*^ mice (Data S1(A, B)).

For further analysis of the extent of inflammation, IFNγ protein levels were measured in the peripheral blood. Without rmTBI, *Hmox1* ^*fl/fl*^ mice and *LyzM‐Cre‐Hmox1* ^*fl/fl*^ mice showed comparable amounts of INFγ (Figure 3K). However, 5 days and 7 days post‐rmTBI, mice with a lack of microglial HO‐1 expression showed elevated INFγ in the peripheral blood (Figure 3L,M).

These data underline the importance of microglial HO‐1 in modulating the inflammatory response in rmTBI.

### 3.4 Wake‐Up Time Post‐rmTBI Can Be Reduced by Treatment With Exogenous CO

**FIGURE 4:**
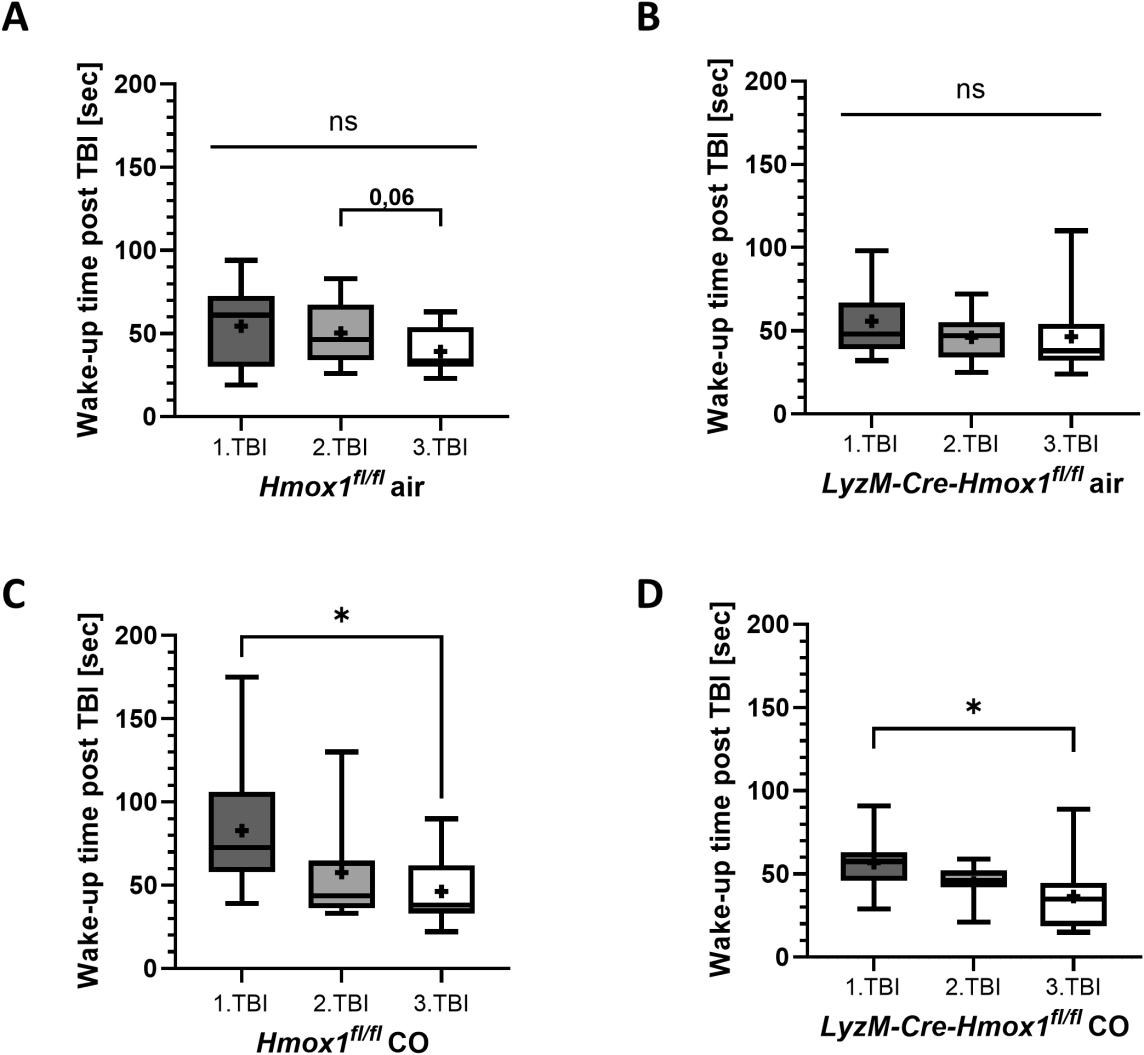
Reduction of wake‐up time post‐repetitive mild traumatic brain injury (rmTBI) by treatment with exogenous CO. Wake‐up time after isoflurane anaesthesia and TBI impact in seconds of *Hmox1* ^*fl/fl*^ and *LyzM‐Cre‐Hmox1* ^*fl/fl*^ mice from the rmTBI model at the time points of the first, second and third mTBI of totally three impacts; ±CO treatment (1 h, 250 ppm daily); *n* = 10–13. (A) *Hmox1* ^*fl/fl*^ air *p* = ns (*F* (DFn, DFd) (1.421, 15.63) = 3.109). (B) *LyzM‐Cre‐Hmox1* ^*fl/fl*^ air *p* = ns (*F* (DFn, DFd) (1.612, 23.37) = 0.7182). (C) *Hmox1* ^*fl/fl*^ CO *p* = 0.192 (*F* (DFn, DFd) (1.921, 21.13) = 4.871). (D) *LyzM‐Cre‐Hmox1* ^*fl/fl*^ CO *p* = 0.022 (*F* (DFn, DFd) (1.838, 16.54) = 4.787). Results presented as bar (mean with SD); statistical analysis used mixed‐effects analysis; statistically significant values were defined as *p* ≤ 0.05 (*p ≤ 0.05); abbreviations: rmTBI = repetitive mild traumatic brain injury, sec = seconds, CO = carbon monoxide.

We previously published the anti‐inflammatory effects of exogenous gaseous CO treatment in mice post‐SAH (Kaiser et al. 2019; Schallner et al. 2015; Kaiser et al. 2024; Henrich et al. 2023). Additionally, as CO is endogenously produced by HO‐1 activity, a lack of HO‐1 is associated with reduced endogenous CO levels. Therefore, we investigated the potential compensation for the lack of endogenous CO production by exogenous CO treatment. The wake‐up time in *Hmox1* ^*fl/fl*^ mice and *LyzM‐Cre‐Hmox1* ^*fl/fl*^ mice without CO treatment did not change throughout the repeated mTBIs. However, with *p* = 0.06, the wake‐up time in *Hmox1* ^*fl/fl*^ mice after the third mTBI was slightly reduced (Figure 4A,B). In contrast, daily treatment with exogenous CO starting after the first mTBI significantly reduced wake‐up times in both genotypes (Figure 4C,D). These data point toward a protective effect of CO post‐rmTBI.

### 3.5 Exogenous CO Treatment Alters Inflammation Post‐rmTBI

**FIGURE 5:**
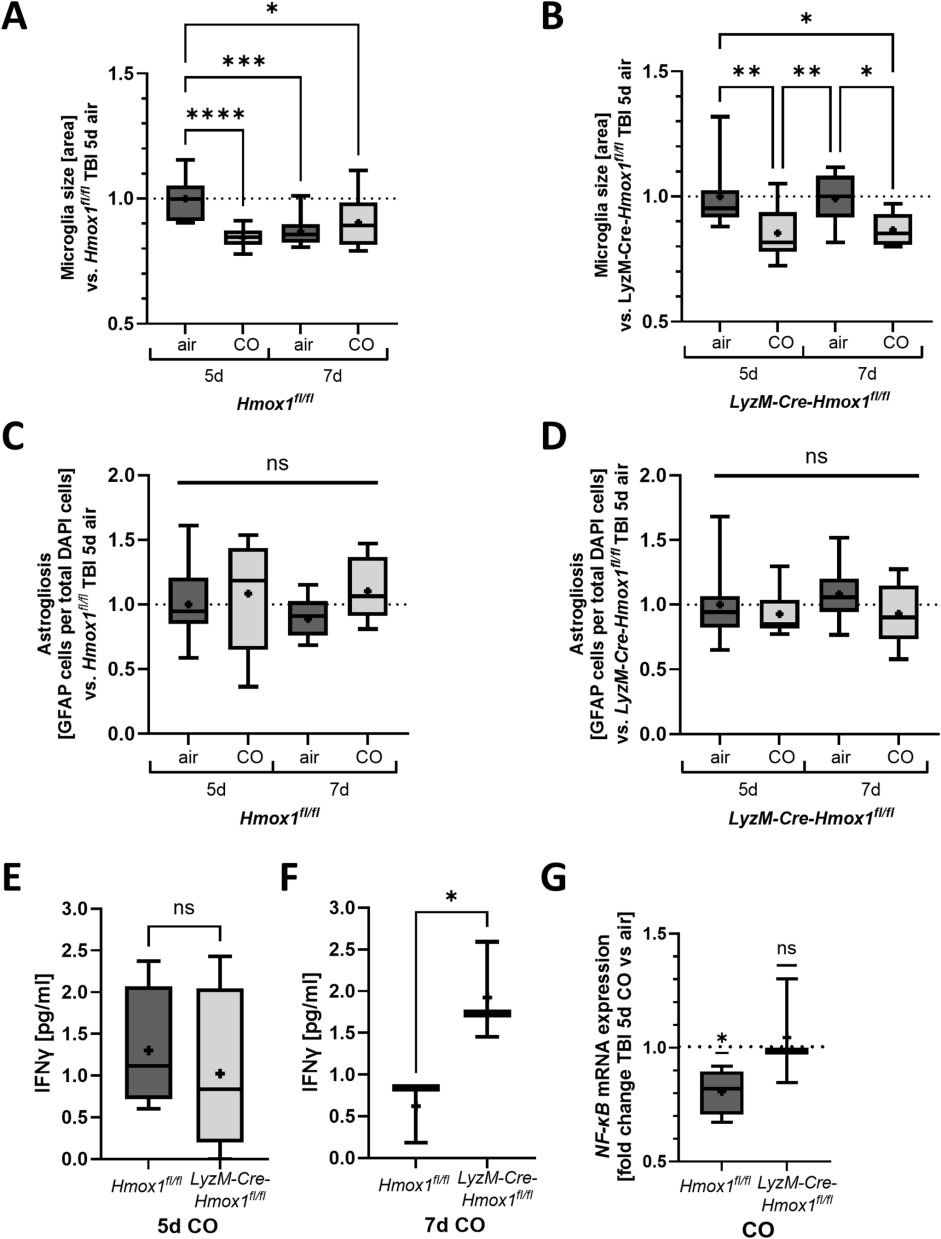
Reduced inflammation post‐repetitive mild traumatic brain injury (rmTBI) by treatment with exogenous carbon monoxide (CO). (A–D) Comparison of *Hmox1* ^*fl/fl*^ and *LyzM‐Cre‐Hmox1* ^*fl/fl*^ mice from the rmTBI model; ±CO treatment (1 h, 250 ppm daily); 5 or 7 days post first impact of rmTBI; calculated by division of all conditions by either *Hmox1* ^*fl/fl*^ or *LyzM‐Cre‐Hmox1* ^*fl/fl*^ 5 days air treated. (A, B) Cortex microglia soma size; *n* = 12 analysed images per group. (A) *p* ≤0.0001 5 days air versus 5 days CO; *p* = 0.0006 5 days air versus 7 days air; *p* = 0.017 5 days air versus 7 days CO (*F* (DFn, DFd) (3, 44) = 9.762). (B) *p* = 0.0052 5 days air versus 5 days CO; *p* = 0.0119 5 days air versus 7 days CO; *p* = 0.0088 5 days CO versus 7 days air; *p* = 0.0194 7 days air versus 7 days CO (*F* (DFn, DFd) (3, 44) = 7.265). (C, D) Quantification of the number of astrocytes in the cortex; measured as GFAP‐DAPI‐double‐positive cells per total DAPI count; *n* = 11–12 analysed images; (C) *p* = ns (*F* (DFn, DFd) (3, 42) = 1.334); (D) *p* = ns (*F* (DFn, DFd) (3, 44) = 1.310). (E, F) Flow cytometric analysis of cytokine IFNγ protein level in peripheral blood of *Hmox1* ^*fl/fl*^ and *LyzM‐Cre‐Hmox1* ^*fl/fl*^ rmTBI mouse model with CO treatment (1 h, 250 ppm daily); 5 (E) or 7 (F) days post first impact of rmTBI; *n* = 3–4; (E) *p* = ns (*F* (DFn, DFd) (3, 3) = 1.815); (F) *p* = 0.0329 *p* = ns (*F* (DFn, DFd) (2, 2) = 2.431). (G) NF‐κB mRNA level in the cortex of *Hmox1* ^*fl/fl*^ and *LyzM‐Cre‐Hmox1* ^*fl/fl*^ mice 5 days post rmTBI with CO treatment (1 h, 250 ppm daily); demonstrated as fold change versus air treated mice; *n* = 3–4 mice; *p* = 0.0321 *Hmox1* ^*fl/fl*^ 5d CO versus 1 (df = 3); *p* = ns *LyzM‐Cre‐Hmox1* ^*fl/fl*^ 5d CO versus 1 (df = 2). Results presented as box blot (whiskers indicate minimum and maximum, the line in the box marks the median and the “+” in the box shows the position of the mean) (A–D) and as bar (mean with SD) (E–G); statistical analysis used ordinary one‐way ANOVA with multiple comparison test (Tukey's) (A–D), unpaired *t*‐test (E, F) and one sample *t*‐test (G). Statistically significant values were defined as *p* ≤ 0.05 (*p ≤ 0.05; **p ≤ 0.01; ***p ≤ 0.001; ****p ≤ 0.0001); abbreviations: rmTBI = repetitive mild traumatic brain injury, d = day; pg/mL = picogram per millilitre, CO = carbon monoxide.

After having confirmed that exogenous CO treatment reduces wake‐up times in both *Hmox1* ^*fl/fl*^ and *LyzM‐Cre‐Hmox1* ^*fl/fl*^ mice following rmTBI, we analysed the state of inflammation in the mouse brain with CO treatment post‐rmTBI. Microglia size, thus their activation state, was reduced in CO‐treated *Hmox1* ^*fl/fl*^ mice 5 days post‐rmTBI (Figure 5A) and in *LyzM‐Cre‐Hmox1* ^*fl/fl*^ mice 5 days and 7 days post‐rmTBI (Figure 5B). On the other hand, CO did not alter the number of astrocytes post‐rmTBI either in mice expressing HO‐1 or in KO mice (Figure 5C,D). While 5 days post‐rmTBI a higher IFNγ expression was observed in KO mice compared to *Hmox1* ^*fl/fl*^ mice (Figure 3L), after CO treatment, this difference was diminished (Figure 5E). On the other hand, the considerably higher expression of INFγ at Day 7 post‐rmTBI (Figure 3M) could also be observed in mice treated with CO (Figure 5F). However, S100B mRNA expression as well as global HO‐1 expression in the cortex post‐TBI were not affected by exogenous CO, neither in *Hmox1* ^*fl/fl*^ nor in *LyzM‐Cre‐Hmox1* ^*fl/fl*^ mice (Data S1(C, D)). Interestingly, exogenous CO caused a reduction in NF‐κB mRNA expression in *Hmox1* ^*fl/fl*^ mice but not in *LyzM‐Cre‐Hmox1* ^*fl/fl*^ mice, implying a role of microglial HO‐1 expression in this process (Figure 5G).

## 4 Discussion

The present study highlights the role of HO‐1 in the inflammatory response and recovery post‐rmTBI. Mice lacking microglial HO‐1 showed a blunted immediate response to the repetitive injury due to a pre‐existing activated state of microglia and astrocytes compared to mice with normal microglial HO‐1 expression. This points toward a higher basal inflammation in the absence of HO‐1 in microglia. Nevertheless, KO mice showed higher and sustained inducibility of NF‐κB and peripheral IFNγ toward later phases of the injury response, showing the varied complex role of HO‐1 in the baseline activation state versus the acute activation state of microglia. Endogenous CO reduced the inflammation and partially compensated for the lack of endogenous CO production via HO‐1.

Induction of HO‐1 is widely known as anti‐inflammatory in many disease models, including TBI (Zhu et al. 2018; Dai et al. 2018; Yang et al. 2016; Shu et al. 2016). This is in line with previously published data, which show that inhibition of microglia activation leads to increased HO‐1 expression, along with the release of anti‐inflammatory cytokines (Subedi et al. 2019; Yang et al. 2020). In this study, we observed microglia activation and astrogliosis at Day 5 post‐rmTBI in HO‐1 KO mice to a similar extent as in mice with normal HO‐1 expression. Nevertheless, we were able to identify underlying differences. The HO‐1 KO in microglia led to a higher increase of NF‐κB expression and IFNγ release compared to *Hmox* ^*fl/fl*^ mice on Day 5, thus further suggesting a regulatory role of HO‐1 with regard to the inflammatory response. Even in the absence of haemorrhage, as in our rmTBI model, the ability to degrade heme plays a crucial role in the anti‐inflammatory mechanisms. Tissue disruption after trauma leads to the release of heme‐containing proteins like the mitochondrial cytochromes (Chang et al. 2005). Heme contributes to oxidative stress and acts as a damage‐associated molecular pattern (DAMP) (Bozza et al. 2020). HO‐1 degrades heme by releasing CO, iron and biliverdin. Biliverdin itself has anti‐inflammatory effects like ROS reduction leading to lower NF‐κB activity (Gibbs and Maines 2007), which is consistent with our data. Additionally, in our study, the inflammatory response had diminished in wild‐type mice by Day 7 post‐rmTBI. In contrast, HO‐1‐deficient mice exhibited sustained neuroinflammation on Day 7, emphasizing the anti‐inflammatory role of HO‐1 in recovery after TBI. Between Day 5 and Day 7, various molecular mechanisms may influence glial activation states in wild‐type mice, including alterations in oxidative stress levels and changes in cytokine release, such as IFNγ. The absence of microglial HO‐1 appears to result in dysregulated inflammation. Additionally, HO‐1 deficiency may reduce the ability of glial cells to transit from proinflammatory (M1‐like) to reparative (M2‐like) states. Indeed, in vitro and in vivo studies with anti‐inflammatory substances show enhanced M2 transition driven by HO‐1 pathway activation (Wang et al. 2022, 2023). In patients, cerebral inflammation post‐TBI has been shown to exacerbate neuronal damage, worsening outcomes (Xiong, Mahmood, and Chopp 2018). Therefore, early reduction of inflammation would be therapeutically beneficial.

In our study, we detected an increased activation state of microglia lacking HO‐1, even in the absence of a damaging impact. However, resting microglia express low levels of HO‐1 (Xia et al. 2023; Lee et al. 2015). Our data suggest a role for HO‐1 in the maintenance of the resting state of microglia, despite their low basal expression levels. Resting microglia must carefully control their state, as they constantly monitor their environment for potential threats or damage while remaining relatively “inactivated” under physiological conditions (Kierdorf and Prinz 2017). Although studies on HO‐1 in this resting context are missing, it is highly likely that its anti‐inflammatory properties help to keep microglia in check in healthy tissue.

TBI leads to the activation of microglia, which release proinflammatory cytokines and chemokines to activate adjacent astrocytes (Zheng et al. 2022; Sofroniew and Vinters 2010; Witcher et al. 2018). A published study using a TBI mouse model demonstrated enhanced astrogliosis following TBI (Witcher et al. 2018). In wild‐type mice, we observed elevated numbers of astrocytes in the cortex. In mice lacking microglial HO‐1, we observed altered astrogliosis at the site of injury and inflammation. Activated microglia release chemokines to regulate astrocyte proliferation and astrocyte migration to the damaged site (Zhang et al. 2010). Changes in cytokine secretion result in disruption of the tightly regulated crosstalk between microglia and astrocytes. In the absence of the protective effects of HO‐1 through anti‐inflammatory bilirubin and CO, the proinflammatory state of microglia activates astrocytes, thereby driving the inflammation toward neurotoxicity.

Vasodilation caused by inflammation can worsen the outcome post‐TBI due to related changes in cerebral macro‐ and microcirculation, which can indirectly affect neuronal damage. In our study, we observed vasodilation post‐rmTBI. The recovery on Day 7 post‐rmTBI was HO‐1 dependent, as vasodilation still occurred in the KO mice on Day 7. This could be caused by the sustained inflammatory response observed in mice lacking microglial HO‐1. Accordingly, we observed higher NF‐κB expression and higher IFNγ protein levels in KO mice compared to wild‐type post‐rmTBI. IFNγ has been reported to enhance microglia activation and worsen neurodegeneration (Zhang et al. 2020). Inhibition of the NF‐κB signal pathway has previously been described as neuroprotective with a reduction of microglia hyperactivation pointing toward a promising therapeutic target point (Wu, Liu, and Guo 2019; Hao et al. 2020).

One limitation concerning our in vivo rmTBI model is the precision of the impact location. Even though precaution was taken to reduce errors, slight differences in the position of the impact on the mouse skull could not be eliminated. Therefore, we focused our analysis on the cortex region closest to the main impact site. Further, it is important to note that our model induced a mild, repetitive brain trauma, typically present in contact sports, and does not present a suitable model to study the mechanisms of inflammation in severe brain trauma. It was important to use mice of the same age and comparable weight and size, as our own preliminary data suggest that this influences the severity of the impact significantly. In general, animal models provide limited insight into processes in humans. Nevertheless, some human studies of the role of HO‐1 in TBI diseases indicate transferability of preclinical data: a publication showed HO‐1‐positive microglia at the side of damage in human TBI (Beschorner et al. 2000), another study on severe TBI in infants displayed significant HO‐1 expression in the cerebrospinal fluid (Cousar et al. 2006), as well as our own previous work analysing patients with delirium, where HO‐1 expression measurement helped predict delirium occurrence post‐TBI (Steimer et al. 2021).

CO was used at nontoxic levels as it has been established in animal and human studies (Otterbein et al. 2000; Stupfel and Bouley 1970). It is still debated if this dose exactly reflects the endogenous amount of CO via HO‐1, but some studies present comparable effects and compensation of endogenous deficiency via exogenous CO application (Schallner et al. 2015; D'Amico et al. 2006). In our study, the first beneficial impact of CO treatment was observed within the time of the third mTBI, as the CO‐treated wild‐type as well as KO mice displayed shortened wake‐up time compared to the air‐treated group. In further analysis, we could confirm that exogenous CO application did reduce microglia activation, which was interpreted as being beneficial, in line with previously published data attributing this to reduced inflammation after neuronal injury (Qin et al. 2023). However, as astrogliosis was not affected by CO treatment in our study, the anti‐inflammatory effect seemed to come mainly from the CO effect directly on microglia. Our prepublished data in SAH mouse models underlines that CO influences the inflammatory effect exerted by microglia (Kaiser et al. 2024; Henrich et al. 2023). However, it has to be considered that HO‐1 was only lacking in microglia, thus reducing the endogenous CO level only in those cells, leading to a higher compensatory effect by exogenous CO, mainly in microglia, which, in contrast to astrocytes, lacked endogenous CO production. To confirm this potential cell‐specific effect of exogenous CO treatment, mice with HO‐1 KO in astrocytes should be investigated in future investigations.

## 5 Conclusion

Our data demonstrate a modulating and protective role of the HO‐1/CO system during the inflammatory response following rmTBI. These findings might improve the understanding of the underlying mechanisms that determine neuronal damage and recovery after brain trauma. In the future, favourably influencing the HO‐1/CO pathway might prove to be of clinical benefit in patients suffering from mild repetitive head trauma.

## Author Contributions

**Sandra Kaiser:** data curation, formal analysis, investigation, methodology, project administration, supervision, validation, visualization, writing – original draft. **Anna Fritsch:** data curation, formal analysis, investigation, validation, visualization. **Lena Jakob:** data curation, formal analysis, investigation. **Nils Schallner:** conceptualization, funding acquisition, methodology, project administration, resources, supervision, validation, writing – original draft.

## Ethics Statement

The animal study protocol was approved by the regional board of Freiburg (protocol code G‐20‐159 [date of approval 18.01.2021]).

## Conflicts of Interest

The authors declare no conflicts of interest.

## Supporting information


**Data S1** S100B and HO‐1 mRNA expression post‐TBI expression levels of mRNA in the cortex of *Hmox1* ^*fl/fl*^ and *LyzM‐Cre‐Hmox1* ^*fl/fl*^ mice either with no TBI (control) or 5 days post rmTBI with or without CO treatment (1 h, 250 ppm daily); demonstrated as fold change; (A) *S100B* mRNA expression; *n* = 4 mice; *p* = 0.0073 *Hmox1* ^*fl/fl*^ versus 1 (df = 3); *p* = 0.0032 *LyzM‐Cre‐Hmox1* ^*fl/fl*^ versus 1 (df = 3). (B) *HO‐1* mRNA expression; *n* = 4 mice; *p* = 0.0385 *Hmox1* ^*fl/fl*^ versus 1 (df3); *p* = 0.0336 *LyzM‐Cre‐Hmox1* ^*fl/fl*^ versus 1 (df = 3). (C) *S100B* mRNA expression; *n* = 4 mice; *p* = ns *Hmox1* ^*fl/fl*^ CO versus 1 and *LyzM‐Cre‐Hmox1* ^*fl/fl*^ CO versus 1. (D) *HO‐1* mRNA expression; *n* = 4 mice; *p* = ns *Hmox1* ^*fl/fl*^ CO versus 1 and *LyzM‐Cre‐Hmox1* ^*fl/fl*^ CO versus 1. Results were presented as box blot (whiskers indicate minimum and maximum, the line in the box marks the median and the “+” in the box shows the position of the mean); statistical analysis used unpaired *t*‐test and one sample *t*‐test; statistically significant values were defined as *p* ≤ 0.05 (*p ≤ 0.05; **p ≤ 0.01); abbreviations: TBI = mild traumatic brain injury, d = day; CO = carbon monoxide.

## Data Availability

The data that support the findings of this study are available from the corresponding author upon reasonable request.
