# Supplementary figures and images for: Severity of Repetitive Mild Traumatic Brain Injury Depends on Microglial Heme Oxygenase‐1 and Carbon Monoxide
Source: Eur J Neurosci. 2025 Jan 22;61(2):e16666. doi: 10.1111/ejn.16666 (PMC11755003; doi:10.1111/ejn.16666)

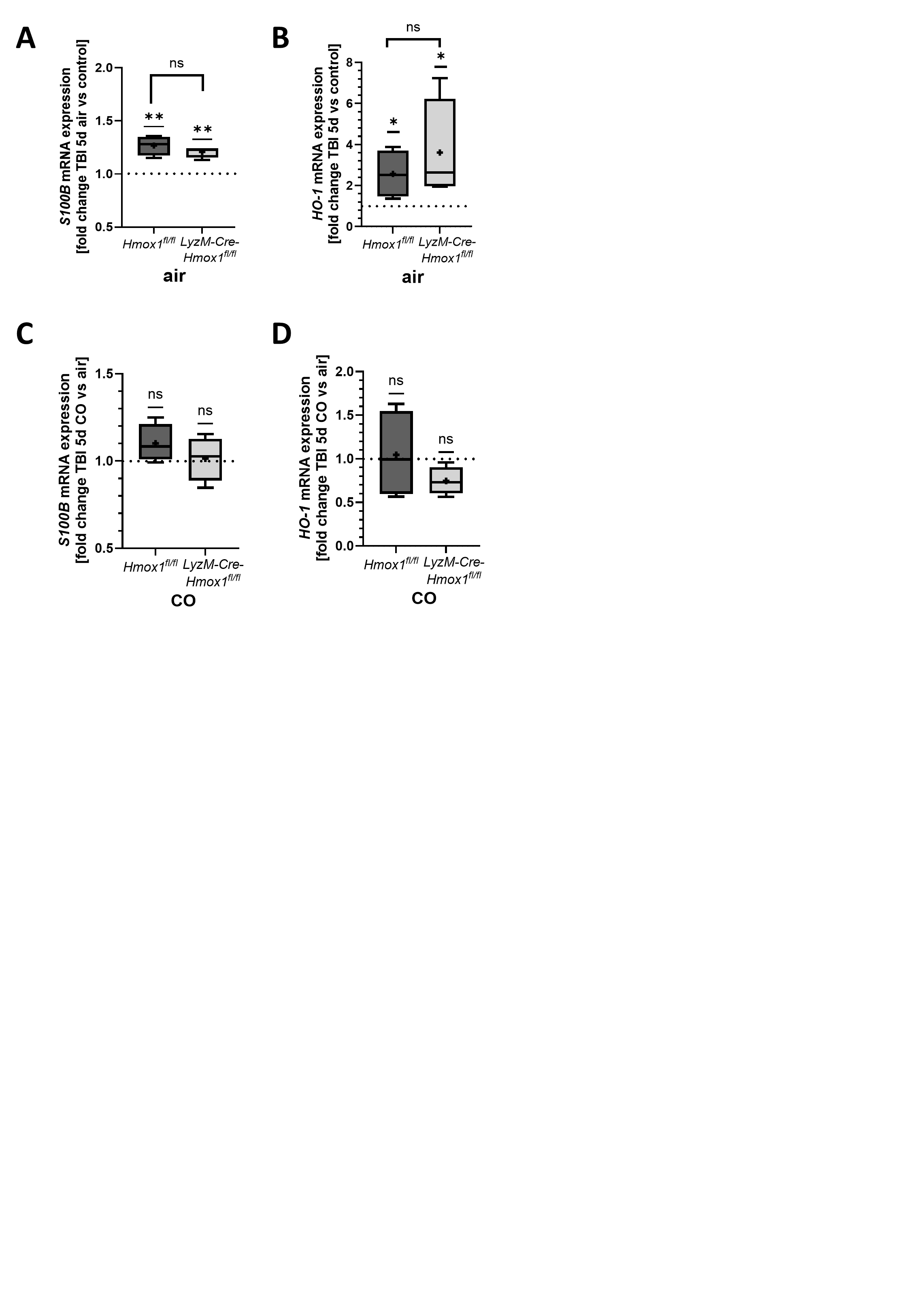

Supplement: Supplementary file 1 — Data S1 S100B and HO‐1 mRNA expression post‐TBI expression levels of mRNA in the cortex of Hmox1 fl/fl and LyzM‐Cre‐Hmox1 fl/fl mice either with no TBI (control) or 5 days post rmTBI with or without CO treatment (1 h, 250 ppm daily); demonstrated as fold change; (A) S100B mRNA expression; n = 4 mice; p = 0.0073 Hmox1 fl/fl versus 1 (df = 3); p = 0.0032 LyzM‐Cre‐Hmox1 fl/fl versus 1 (df = 3). (B) HO‐1 mRNA expression; n = 4 mice; p = 0.0385 Hmox1 fl/fl versus 1 (df3); p = 0.0336 LyzM‐Cre‐Hmox1 fl/fl versus 1 (df = 3). (C) S100B mRNA expression; n = 4 mice; p = ns Hmox1 fl/fl CO versus 1 and LyzM‐Cre‐Hmox1 fl/fl CO versus 1. (D) HO‐1 mRNA expression; n = 4 mice; p = ns Hmox1 fl/fl CO versus 1 and LyzM‐Cre‐Hmox1 fl/fl CO versus 1. Results were presented as box blot (whiskers indicate minimum and maximum, the line in the box marks the median and the “+” in the box shows the position of the mean); statistical analysis used unpaired t‐test and one sample t‐test; statistically significant values were defined as p ≤ 0.05 (*p ≤ 0.05; **p ≤ 0.01); abbreviations: TBI = mild traumatic brain injury, d = day; CO = carbon monoxide. [file EJN-61-0-s001.tif]
